# Symptomatic Neovaginal Candidiasis in Transgender Women After Penile Inversion Vaginoplasty: A Clinical Case Series of Five Consecutive Patients

**DOI:** 10.1089/trgh.2017.0045

**Published:** 2018-05-01

**Authors:** Kristin B. de Haseth, Marlon E. Buncamper, Müjde Özer, Lian Elfering, Jan Maerten Smit, Mark-Bram Bouman, Wouter B. van der Sluis

**Affiliations:** ^1^ Department of Plastic, Reconstructive and Hand Surgery, VU University Medical Centre, Amsterdam, The Netherlands.; ^2^ EMGO Institute for Health and Care Research, VU University Medical Centre, Amsterdam, The Netherlands.; ^3^ Centre of Expertise on Gender Dysphoria, VU University Medical Centre, Amsterdam, The Netherlands.; ^4^ Gender Surgery Amsterdam, Amsterdam, The Netherlands.

**Keywords:** Candida, candidiasis, gender dysphoria, transgender, vaginoplasty

## Abstract

**Background:** Vaginoplasty is performed as gender-affirming surgery in transgender women. While multiple surgical techniques exist for this goal, penile inversion vaginoplasty is performed most frequently. Neovaginal symptoms may impede sexual functioning after surgery.

**Methods:** A total of five consecutive patients with symptoms and positive swabs for neovaginal candida infection were described.

**Results:** All patients presented with white-colored neovaginal discharge and some with neovaginal itching and/or malodor. All were topically treated with miconazole, which resulted in symptom clearance. Follow-up swabs were negative for candida.

**Conclusions:** To our knowledge, this is the first report on (symptomatic) candidiasis of the penile-inverted neovagina.

## Introduction

Vaginoplasty, the surgical creation of a vagina, is performed as vaginal reconstruction in nontransgender women with vaginal absence or as gender-affirming surgery in transgender women. While different surgical techniques exist for the latter group, penile inversion vaginoplasty is the most frequently performed technique.^1^ During surgery, most of the penis is disassembled and the components are used for vulvar, vaginal, and clitoral construction.^2^ A neovaginal cavity is dissected in Denonvilliers' space between the bladder and rectum. This neovaginal cavity is lined with inverted penile skin, which produces a skin-lined vaginal cavity that shrinks little, is nonhair bearing, and sensate. The goal of vaginoplasty is to create a feminine vulva, a hooded and sensate clitoris, labia minora, and, for those who express the wish to be able to engage in neovaginal penetrative intercourse, a deep and wide enough neovaginal cavity to facilitate penetration, all with the least possible surgical complications.^3^ Before surgery, the genital is generally depilated. In the first days after penile inversion vaginoplasty, the neovagina is rinsed with betadine, but later only with water. Dilation is performed twice a day in the first postoperative year. Dilators are routinely cleansed with water, but not regularly with antimicrobial agents. After surgery, sexual functioning and postoperative satisfaction are paramount. Little is known on microbiological and fungal characteristics or associated pathology of the neovagina. Bacterial and/or fungal overgrowth or infections of the neovagina may cause significant discomfort to individual patients and may lead to sexual dysfunction, especially in a relationship with a sexual partner. In this study, we describe five consecutive patients with symptomatic neovaginal candidiasis.

## Materials and Methods

Five consecutive patients with neovaginal candidiasis are described. Patient characteristics, (neovaginal) symptoms, microbial data, and treatment outcomes were recorded. Approval for this study was provided by the local medical ethics committee (METC, Medisch Ethische Toetsings Commissie). All patients provided written consent for use of their (anonymized) data in this article.

## Results

An overview of included patients is presented in Table 1.

**Table 1.** **Overview of Identified Patients with Symptomatic Neovaginal Candidiasis**

Case No.	Age at surgery	Postoperative time at presentation (months)	Somatic comorbidity	Psychiatric comorbidity	Medication	BMI (kg/m^2^)	Intoxications	Sexual activity	Symptoms	Clinical follow-up (months)
1	29	12	—	—	Estrogen	22.5	—	No sexual activity	Neovaginal discharge	13
									Neovaginal itching	
									Pain during dilatation	
2	45	20	Inactive hepatitis C	—	Estrogen	27.8	—	No sexual activity	Crumbly white discharge	65
									Unpleasant neovaginal odor	
									Vulvar itch	
3	29	14	Epilepsy	Depression	Estrogen, phenytoin, tianeptine	30.4	10 cigarettes per day	Sexually active with >10 male partners in the last 3 months	White-colored neovaginal discharge	24
4	61	1	Epilepsy, diabetes	—	Estrogen, metformin	27.1	—	—	White-colored neovaginal discharge	36
									Neovaginal itching	
5	24	3	Asthma	Depression, PTSS, eating disorder	Estrogen	26.9	5 cigarettes per day	Sexually active with >10 male partners in the last 3 months	White-colored neovaginal discharge	30

BMI, body mass index; PTSS, post traumatic stress syndrome.

### Case descriptions

#### Case 1

A 30-year-old transgender woman of Dutch origin, who underwent penile inversion vaginoplasty 12 months before presentation, consulted our outpatient clinic with symptoms of neovaginal discharge, neovaginal itching, and pain during dilation. She had been using estrogen as hormonal treatment for 4 years. She rinsed her vagina biweekly with water without soap or other products. There was no comorbid somatic illness or obesity. She did not use alcohol, tobacco, or drugs. She had not engaged in penetrative sexual intercourse after surgery, because she had not found a sexual partner at that point. At examination, mild pelvic floor hypertonicity was noted. An atrophic neovaginal skin lining was observed with whitish-gray discharge in the neovaginal cavity. Fungal culturing revealed the presence of Candida species. She was topically treated with miconazole vaginal cream (20 mg/g), which induced symptom relief within 2 weeks. At follow-up visits, no neovaginal abnormalities were observed.

#### Case 2

A 47-year-old transgender woman of Dutch origin, who underwent penile inversion vaginoplasty 20 months before, consulted our outpatient clinic with complaints of crumbly white discharge, an unpleasant neovaginal odor, and a vulvar itch. She had been using estrogen as hormonal therapy for 3 years. She did not use alcohol, tobacco, or drugs. She had comorbid hepatitis C. At neovaginal examination, white discharge was observed in the neovaginal cavity and at the vulva. Fungal culturing revealed the presence of Candida species. She was topically treated with miconazole vaginal cream (20 mg/g), which induced symptom relief in a week. At 65 months of clinical follow-up, no recurrence was observed.

#### Case 3

A 30-year-old transgender woman of Dutch origin, who underwent penile inversion vaginoplasty combined with a bilateral breast augmentation with prosthesis, 14 months before, consulted our outpatient clinic with complaints of white-colored neovaginal discharge. She had been using estrogen as hormonal treatment for 6 years. She did not use alcohol or drugs, but smoked 10 cigarettes per day. She had comorbid epilepsy and depression, for which she was treated with phenytoin and tianeptine. She had been sexually active with more than 10 male partners in the last 3 months and did not engage in sex work. At neovaginal examination, whitish cottage cheese-like discharge and some hypergranulation were observed in the neovaginal cavity. Neovaginal fungal culturing revealed the presence of Candida species. Urine cultures were negative for Candida. She was topically treated with a miconazole 1200 mg vaginal capsule, which induced symptom relief. With 24 months of clinical follow-up, there was no recurrence of symptoms.

#### Case 4

A 61-year-old transgender woman of Dutch origin, who underwent penile inversion vaginoplasty combined, 1 month before, consulted our outpatient clinic with complaints of white-colored neovaginal discharge. She had comorbid diabetes, for which she used metformin. At neovaginal examination, whitish discharge was observed in the neovaginal cavity. Fungal culturing revealed the presence of *Candida glabrata*. She was treated with a miconazole 1200 mg vaginal capsule, which induced symptom relief. With 36 months of clinical follow-up, there was no symptom recurrence.

#### Case 5

A 25-year-old transgender woman of Asian origin, who underwent penile inversion vaginoplasty 3 months before, consulted our outpatient clinic with white neovaginal discharge. She worked in the commercial sex industry, was HIV-negative, and engaged in sexual intercourse with more than 10 males in the last 3 months. The sexually transmitted disease workup revealed no presence of STDs. Fungal culturing revealed the presence of *Candida albicans*, which was treated with miconazole vaginal cream (20 mg/g), inducing symptom relief in a week. At 30 months of clinical follow-up, there was no symptom of recurrence.

## Discussion

In this study, five patients with symptomatic neovaginal candidiasis after penile inversion vaginoplasty are described. They presented with neovaginal discharge, an unpleasant odor, and/or an itchy sensation. All were treated topically with miconazole, which induced symptom clearance in all.

Currently, no data exist on the effectiveness of topical versus systemic treatment of neovaginal candidiasis in this patient population. The surgically created neovagina, and its vascularization, is structurally different to the vagina of nontransgender women.^4^ Choosing a topical treatment, such as miconazole as cream or vaginal capsule, as first treatment option seems just in this patient population. The use of a vaginal capsule seems preferable, because it can be easily applied and does not interfere with the neovaginal rinsing regimen.

In a Belgian study, smears from the penile-inverted neovagina were obtained from 50 transgender women.^5^ Candida cells were not observed in any of the smears. To our knowledge, this is the first report on candidiasis in the penile-inverted neovagina. Candidiasis is regarded the most common cause of vaginitis after bacterial vaginosis in native women. Approximately 85% of cases are caused by *C. albicans.*^6^ Candida can be present in asymptomatic women as well and its prevalence is estimated to be ∼20%.^7^ Certain host and environmental factors predispose for vaginal candidiasis. Patient-related risk factors include pregnancy, diabetes mellitus, recent use of antibiotics, and immunocompromising comorbidities, such as AIDS.^8^ Whether the same factors contribute to candidiasis of the surgically created neovagina remains to be seen. The intestine is frequently mentioned as reservoir for vaginal colonization by Candida species.^9^ The same has been hypothesized for intestinal microorganisms and surgically formed neovagina to host intestinal microorganisms.^10^

Discharge of the penile-inverted neovagina may be caused by sexually transmitted diseases, candidiasis, hypergranulation, skin or skin graft necrosis, microbial dysbiosis, poor neovaginal hygiene, or rectoneovaginal fistulas. Although neovaginal candidiasis does not seem to be a major health problem in transgender women, it may cause significant discomfort to individual patients, who subsequently seek professional medical care. Healthcare workers associated with transgender patients need to be aware of the condition.

## Abbreviation Used

BMI: body mass index
METC: Medisch Ethische Toetsings Commissie
STD: sexually transmitted disease
